# The Province and Duties of the Dentist

**Published:** 1866-01

**Authors:** H. J. Cressinger


### THE PROVINCE AND DUTIES OF THE DENTIST.

Read before the Central Ohio Dental Association, at Wooster, Nov. 14, '65

BY H. J. CRESSINGER.

MAN'S wants are as varied as his physical organization is wonderful, and he who knows and understands them best, can best supply them. From the day of his birth to the day of his death, is he a slave to that which sustains existence. But, it is our aim to speak only of wants necessary to his happiness, which are pertinent to us as a profession.

That the amount of study and practice necessary to become a proficient in dentistry, is too little appreciated, none will deny. True, a man who has naturally a high order of endowments may originate that which is valuable. Such can spring only from the few of the indomitable zeal and ambition of Drs. Franklin, Watt, and men of like ilk; yet, such instances cannot be regarded as arguments against systematic training. Nature does not make a man a dentist. He may have superior gifts for such a calling, but he must become acquainted with the agencies which he employs, else his efforts must be experiments, nothing certain; whereas, a familiarity with principle and laws will enable him to call to his aid instrumentalities the ignorant know not of.

Let me not be understood, that I disregard the honorable efforts of those who have never had educational advantages, far from it; neither would I have you grope on, year after year, in "the good old way," following a routine you have not the moral courage to change, or, adhering to with willful obstinacy and prejudice, simply to be revenged upon, perhaps, your brother competitor.

Labor and perseverance, in as honorable calling as ours, will be rewarded; it is noble; but a bickering, biased, prejudiced disposition, will *drag its possessor downward.* When I speak of attainments and advantages in dental education, I do not limit it to the class who are situated pecuniarily that they can finish a course of study and graduate. Now, we want theory, we want science, but they will avail us nothing unless accompanied with practical and hard work. It is one thing to direct, but quite another to accomplish.

It is not practical for all to become eminent, but we can labor each in his own sphere and be useful. We need not sit down contented, because we have a large practice, and say, "well, all I have to do is to work away, and become rich," and how often is it remarked by those outside of the profession, "what an easy time Dr. A. has of it! How much money he makes!" True, a dentist having a large patronage, makes money — and he ought to, for in nine cases out of ten he has worked hard for it; and I must say to you members of this association that, though I have had less experience than most of you, I have never yet been able to find the "easy times" so much talked about. On the contrary, it is anything but an easy task, to perform successfully a difficult dental operation. It requires a firm and unflinching nerve, and good physical endurance to labor successfully at all in our calling. And after years of study and labor, who among us is satisfied with the work of his hands? How many imperfections we see — desire is not satisfied, rest is not found. There are those who are so self-sufficient as to flatter themselves that their productions are perfect. To such, an appeal would be folly — for egotism has dammed up the spring of aspiration, and ignorance holds unlimited sway.

I shall advert to but few of the duties of the dentist. And first, the mechanical department claims our attention, and here a *decided* and *persevering* taste for the fine arts should occupy the student's mind.

An error is probably committed by most dentists, with students, in putting them upon artificial work before they have learned one principle or law involved in the work. If it be the first work a student can perform, it does not follow that he undertake it before at least a moderate knowledge is attained. As soon place the inexperienced helmsman at his post in a floundering sea, instead of a calm, and expect equal results. Would not such a course tend to retard further effort?

There are technicalities in inserting sets of teeth, that can be learned only through experience. Theory may point the way, but the student must see the application by a competent workman, and then by perseverance, success will reward the student. The manner of taking an impression, of grinding the teeth, adapting them to the model, packing, vulcanizing, and last, but not least, putting them in the mouth of the patron, all require attainments which none but the skillful can hope for in order to succeed.

In the operative department, or "chair-work" as it is sometimes called, we have far greater range. No part of the profession requires so much study and application, to insure success; and it is the experience of all, that daily practice adds to proficiency, that daily we have cases taxing our skill to the utmost — something different from what we have yet seen. The labor also required in this department is trying and wearing on the nerves, and general health of the operator. Inhaling the breath of the patients; the position during the operation; the constant mental strain; being intent upon your work, and yet avoid wounding the patient, all, make it one of the most difficult and arduous accomplishments of the arts. Then how important, that all knowledge which will facilitate such an operation, should be sought for.

As a practical hint, let me call your attention to the condition of your excavators and drills. They should be kept sharp. All have experienced the marked difference between a drill that has been used a week, and one entirely new. Much time (which is always an important consideration with dentists) will be saved, and much unnecessary fatigue to the patient. Little difficulty will be experienced now in procuring good instruments; and for daily operations in filling, the supply of drills and excavators should be constant, at least every week. For fang fillings we have labored under some disadvantages on account of insufficient and unworthy instruments — but that deficiency is now met in Palmer's sets for fang fillings. Your gold is another object of constant care and attention. I can fully attest to the result of using gold that has lost its adhesive properties by age or exposure, and most of you have doubtless experienced the difference in the gold of the same book, the first used working nicely; but as it becomes exposed even a few days, will become dry and brittle, and is very hard to pack or even introduce into the cavity. It should be as ductile and adhesive as possible, and gold foil deficient in these respects is worthless for good operations. Much annoyance and actual difficulty are experienced by many in filling cavities exposed to flooding by saliva, and I am not loth to confess, that it is to me the greatest hindrance I have to contend with in the use of adhesive foil. I have read of inundated fillings, but I am still sceptical, and will continue to be, until I learn by what philosophy adhesive foil can be united, when submerged in water. I have always been taught, that a perfect plug could not be made, unless the walls of the cavity, and the gold were kept perfectly dry. My experience confirms the doctrine, and more ; even the breath will sometimes destroy the unison of particles of foil. There are few plans proposed to remedy the evil, and most of these ineffectual, and with a single operator impracticable. We wait for some inventive genius to extricate us from the dilemma. Begging your leniency for this digression, I resume the subject.

Extraction of the teeth we pass, with the observation that the improvements in extracting instruments render it almost a minor consideration, except, probably, in case of anamolies of the parts, which an intelligent operator will readily overcome.

We notice next, pathological condition, as relating to dentistry, and remark, that here, almost a new field has presented itself within the last few years. The generality of dentists, knew nothing of Physiology and Anatomy, consequently did not attempt treatment of these conditions, and if at all, only at random.

But, time and opportunity have given us light, and now, treatment of all diseases of the mouth, may rightly be assumed by the dentist. There is, probably, no one part of the body, the treatment of which requires such an extensive knowledge of the system, as the mouth and its attendants.

Indeed, the tongue, the gums, the internal membrane of the mouth, are, all more or less affected by every disease humanity is heir to. Then who will deny, that before a correct diagnosis is attained, at least a limited knowledge, further than the immediate parts should be possessed by the dentist. Many are the causes that produce caries, and it is for us to determine what is the immediate cause in such a particular case. When the treatment required is general, the patient may be referred to a physician; but there are many cases in which the dentist may successfully officiate, and indeed, in which it would be *his duty*, especially, when pernicious influences are being excited upon the teeth.

In a word, it is the duty of the dentist, to treat all the parts that surround the teeth and are influenced by them, or that they influence.

To attempt to direct the application of treatment, in any sense, would be entirely out of place here, as we pass to notice a few general principles which should actuate us. We should continually strive to advance, to improve, and I rejoice to say to you that the way is entirely clear. It is open, and free to all, and not alone is it a privilege, but a duty, for *all to labor zealously for higher attainments.* If we would secure even the necessities of life in our avocation, say nothing about affluence, we must labor for *proficiency.* If we would secure an identity in society, we must labor, so that the work we do prove a benefit and a blessing. Let it not be said that "our patients were the victims of our ignorance." Guilt will surely follow, and it ought to, when so much light stares us in the face. Give them rather the benefit of a studied judgment. Labor to alleviate pain and increase happiness. These dental operations (aside from necessarily painful ones,) will not be considered barbarous and hideous as they so often are. Then will the hearty shake of the hand, and the smiling countenance of benefited patients, aid in making ours a *pleasant* rather than an *onerous* calling.

And, in conclusion, let me urge upon you the importance of *co-operating*, of *associating* our efforts for improvement. Cultivate a great degree of *sociability.* Get down off your high horse, *self-sufficiency.* Throw aside *prejudice.* Banish the idea that you have a secret and are going to get rich upon it. *Honor and eminence never reward such a course.* If you are the fortunate owner of a valuable improvement tell it to your brother dentist, and let it benefit him, and thereby benefit your profession; for it is a noble one, and worthy of all your efforts to secure its eminence. Accept a responsibility, and act upon it; for he who would fill the demands made upon the dentist in one day, must embody a versitality of knowledge and skill required by few other professions. We have chosen it as *our profession.* Let us see to it that we assume nothing. Let us become masters of our situation.

ASHLAND, Oct. 31, 1865. N. J. C.

